# Ontogenetic asymmetry modulates population biomass production and response to harvest

**DOI:** 10.1038/ncomms7441

**Published:** 2015-03-04

**Authors:** Birte Reichstein, Lennart Persson, André M. De Roos

**Affiliations:** 1 Department of Ecology and Environmental Science, Umeå University, 90187 Umeå, Sweden; 2 Institute for Biodiversity and Ecosystem Dynamics, University of Amsterdam, PO box 94248, 1090 GE Amsterdam, The Netherlands

## Abstract

Patterns in biomass production are determined by resource input (productivity) and trophic transfer efficiency. At fixed resource input, variation in consumer biomass production has been related to food quality, metabolic type and diversity among species. In contrast, intraspecific variation in individual body size because of ontogenetic development, which characterizes the overwhelming majority of taxa, has been largely neglected. Here we show experimentally in a long-term multigenerational study that reallocating constant resource input in a two-stage consumer system from an equal resource delivery to juveniles and adults to an adult-biased resource delivery is sufficient to cause more than a doubling of total consumer biomass. We discuss how such changes in consumer stage-specific resource allocation affect the likelihood for alternative stable states in harvested populations as a consequence of stage-specific overcompensation in consumer biomass and thereby the risk of catastrophic collapses in exploited populations.

## Abstract (web summary)

The effect of intraspecific body size variation on the efficiency with which energy is transferred between trophic levels is not well understood. Here, Reichstein *et al*. show that biasing resource delivery toward less efficient consumer life stages can lead to a doubling of consumer biomass.

## Graphical abstract

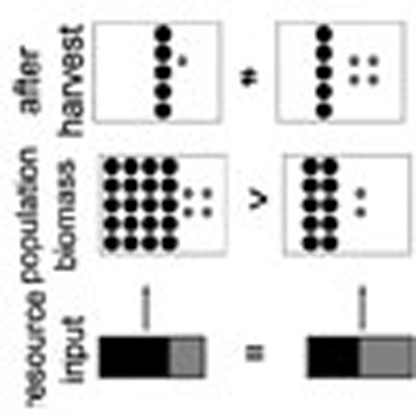

Biomass production, trophic structure and community stability are determined by the efficiency of energy transfer between trophic levels (trophic transfer efficiency) and resource input^1^,^2^,^3^. At constant resource input, biomass production can only be increased if trophic transfer efficiency increases. Overall, the vast majority of studies have concluded that a change in trophic efficiency and, as a result, a change in biomass production can be achieved by altering resource type (quality), consumer metabolic type (for example, ectotherm versus endotherm) or consumer diversity (number of species)^3^,^4^,^5^,^6^,^7^,^8^,^9^,^10^,^11^,^12^,^13^,^14^,^15^,^16^,^17^. In contrast, effects of intraspecific variation on trophic transfer efficiency have been largely ignored. Even studies considering intraspecific variation in terms of genetic variation^17^ neglect the obvious fact that individuals of the overwhelming majority of taxa on Earth grow substantially in size over their life cycle, and that differences in energetic efficiency between life stages potentially have major effects on both population, community and ecosystem dynamics^18^,^19^,^20^. Short-term experiments have in fact demonstrated that the impact of individual organisms on community structure and ecosystem processes can differ more among developmental stages within a species than between species^20^.

For the first time, we here provide long-term experimental evidence that differences in energetic efficiency between juveniles and adults within one population in itself have an impact on the trophic transfer efficiency, and thereby standing biomass and biomass production. Differences between juveniles and adults in energetic efficiency because of different body size scaling of intake rate, metabolic rate or mortality rate can lead to differences between maturation rate and reproduction rate that affect overall population regulation and structure^21^. Theory predicts that when the reproduction rate exceeds the maturation rate a population is controlled by a bottleneck in maturation and becomes dominated by juvenile biomass. Conversely, if the maturation rate exceeds the reproduction rate a population is controlled by a reproduction bottleneck and becomes dominated by adult biomass. Maturation and reproduction rates are not only, as mentioned above, determined intrinsically through stage-specific energetic efficiencies but are also determined extrinsically through stage-specific resource allocation or predation. Differences between these rates have been shown to affect the community structure by causing alternative stable states through emergent Allee effects, emergent facilitation among predators and productivity-driven predator extinctions^21^,^22^,^23^,^24^,^25^,^26^,^27^,^28^,^29^.

We experimentally manipulated the fraction of the overall resource input allocated to juvenile and adult stages in a consumer population while keeping total resource input and food-quality constant. Measured effects included total and stage-specific (juvenile and adult) biomass, maturation and reproduction rate, and stage-specific biomass responses to stage-independent harvest (0.0, 0.01 and 0.017 per capita per day) imposed on a weekly basis. Our study organism was the Least Killifish (*Heterandria formosa*), which is known to be controlled by limited reproduction and is dominated by adult biomass when juveniles and adults share a single resource^25^. Population dynamics were followed over six generations, while the resource input was either allocated equally between the juvenile and the adult stage (1:1) or such that one-third was allocated to the juvenile stage and two-thirds were allocated to the less-efficient adult stage (1:2). Specifically, our experimental system mimicked an ontogenetic habitat shift between juvenile and adult stages where food quality was constant among stages, but stage-specific resource allocation was varied. We chose to mimic an ontogenetic habitat shift because the overwhelming majority of all taxa on Earth go through ontogenetic habitat shifts^30^,^31^ including the majority of insects, amphibians and fish. Furthermore, our experimental design also allowed us to study the sole effect of stage-specific energetic efficiency while keeping aspects such as resource type and consumer species constant.

We show that reallocating a fixed resource input between two consumer stages from an equal resource delivery to juveniles and adults to an adult-biased delivery more than doubles consumer biomass. We then discuss how consumer stage-specific resource allocation affects the occurrence of stage-specific overcompensation in harvested populations and consequently the risk for collapses of exploited populations.

## Results

### Population biomass

**Figure 1:**
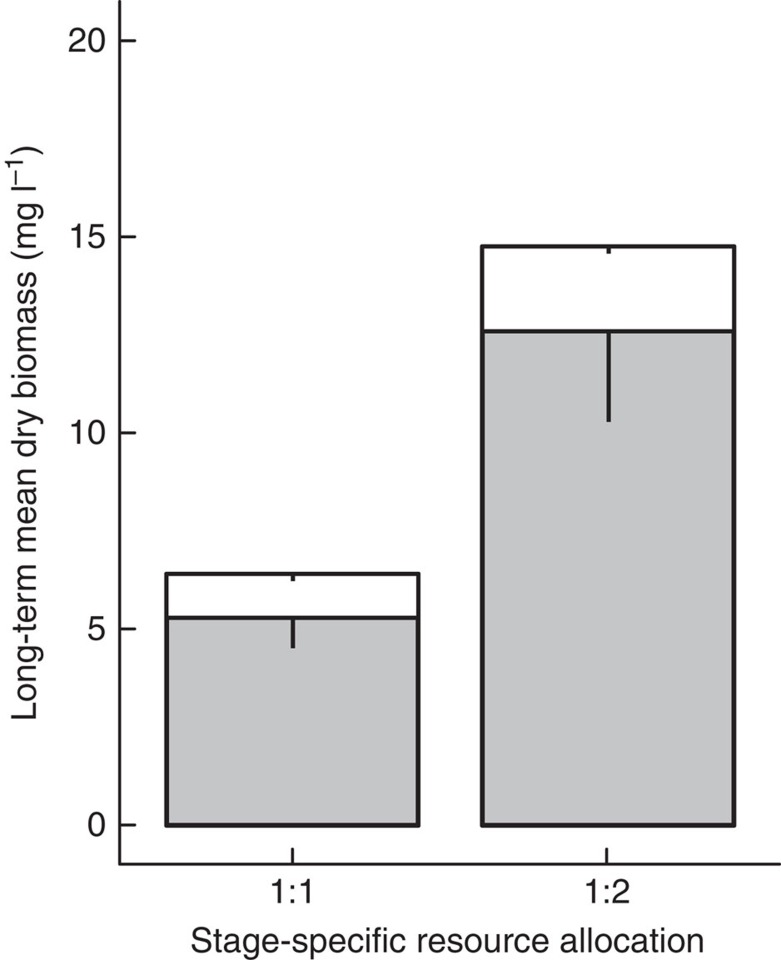
Least Killifish biomass, no harvest applied. Experimental asymptotic long-term average juvenile (white) and adult (grey) Least Killifish dry biomass (mg l^−1^) over six generations for resource input allocated equally between juvenile and adult habitat (1:1) and two thirds resource input allocated to the adult habitat (1:2). Bars represent averages over replicates with vertical lines indicating -1 s.e.m. All treatments were replicated four times.

In Least Killifish populations, in which juveniles and adults inhabited separate habitats, we found that the total population biomass (asymptotic long-term average over the last 32 of 44 weeks) was 2.3 times higher when one-third of the total resource input was allocated to the juvenile habitat and two-thirds to the adult habitat compared with when the system productivity was equally allocated between the two stages (Table 1, Tukey’s honestly significant difference (HSD) test (*n*=4), *P*<0.001; Fig. 1). The mean long-term population biomasses in both resource allocation treatments were dominated by adult biomass, with an approximate adult to juvenile biomass ratio of 5:1. The observed biomass increase resulted from an increase in the biomasses of both stages where adult biomass increased from 5.3 (mean±0.76 s.e.m.) mg l^−1^ to 12.6 (mean±2.25 s.e.m.) mg l^−1^ (Tukey’s HSD test (*n*=4), *P*=0.002), while juvenile biomass increased from 1.1 (mean±0.12 s.e.m.) mg l^−1^ to 2.2 (mean±0.21 s.e.m.) mg l^−1^ (Tukey’s HSD test (*n*=4), *P*=0.066).

### Maturation and reproduction

**Figure 2:**
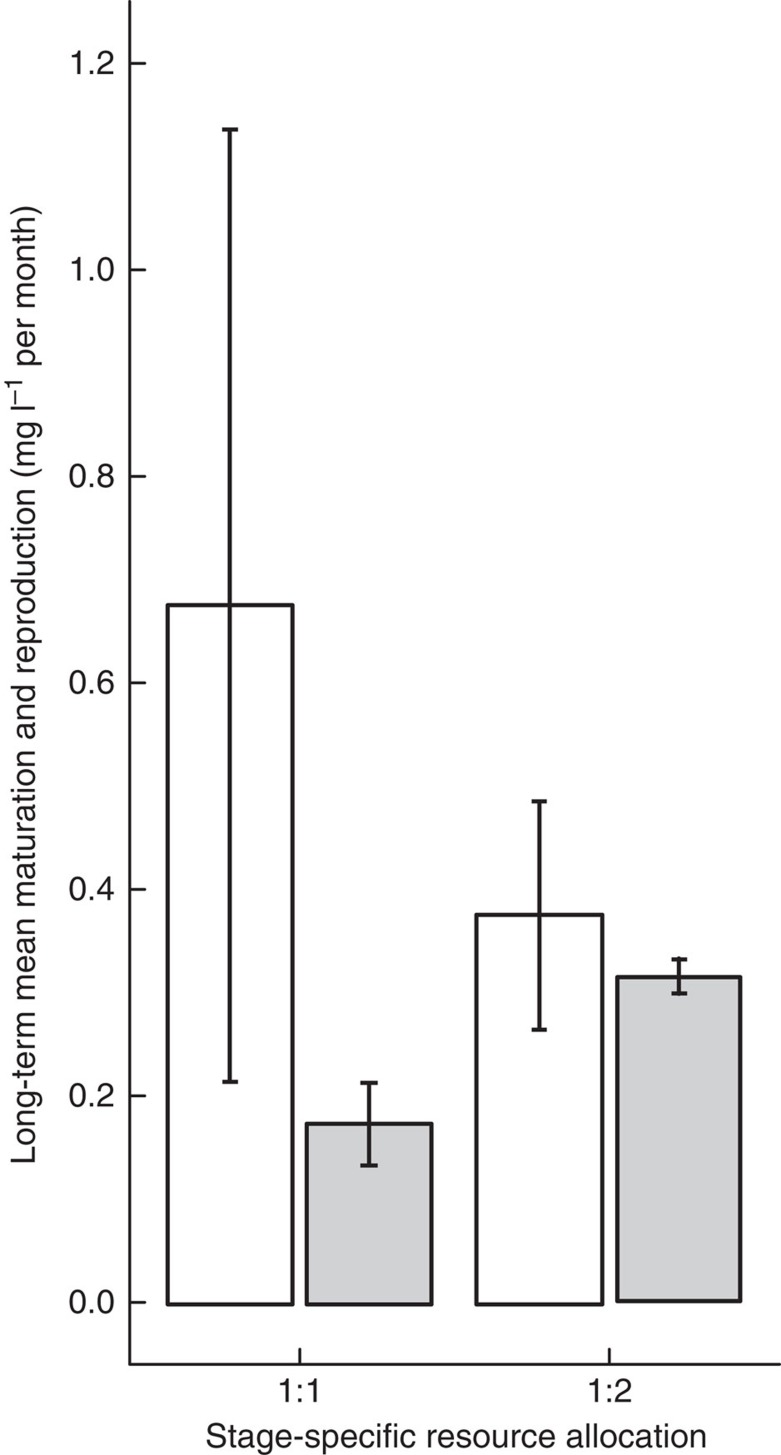
Least Killifish maturation and reproduction, no harvest applied. Experimental asymptotic long-term average monthly Least Killifish reproduction (grey) and maturation (white) rates (mg l^−1^) over six generations for resource input allocated equally between juvenile and adult habitat (1:1), and two thirds resource input allocated to the adult habitat (1:2). Bars represent averages over replicates with error bars indicating ±1 s.e.m. All treatments were replicated four times.

With an equal proportion of total resources allocated to juveniles and adults, the maturation rate was substantially larger than the reproduction rate showing that the system in this case was strongly limited by reproduction (Fig. 2). With equally allocated resource input, adult maintenance costs were relatively high in relation to the available resource, leaving little energy for reproduction and somatic growth. Allocating more of total resource input to the adult stage had a positive effect on reproduction rate; it increased from 0.17 (mean±0.04 s.e.m.) mg l^−1^ per month to 0.32 (mean±0.016 s.e.m.) mg l^−1^ per month, but had no effect on the maturation rate (Table 1; Fig. 2). The mean reproduction rate to maturation rate ratio thereby changed from 1:3 to 1:1. Hence, a resource reallocation to the less-efficient stage led to that the population switched from being limited by reproduction to being limited by maturation and reproduction to the same extent. Intriguingly, this situation with co-limitation of reproduction and maturation (ontogenetic symmetry^18^) coincided with the situation with the higher total consumer biomass (Fig. 1).

### Biomass response to stage-independent harvest

**Figure 3:**
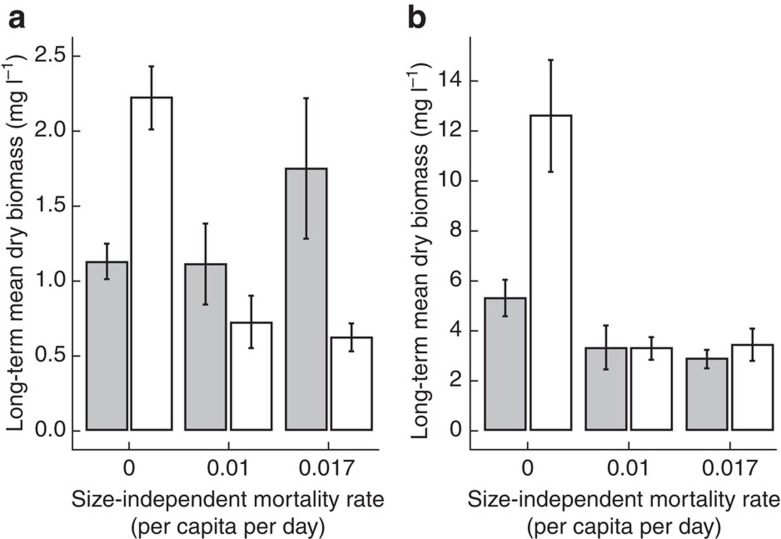
Least Killifish biomasses for all harvest treatments. Juvenile (**a**) and adult (**b**) Least Killifish asymptotic long-term average dry biomass (mg l^−1^) over six generations under three stage-independent harvest regimes (0.0, 0.01, and 0.017 per capita day^−1^) for resource input equally allocated between the juvenile and adult habitat (grey) and two thirds resource input allocated to the adult habitat (white). Bars represent averages over replicates with error bars indicating ±1 s.e.m. All treatments were replicated four times.

Juvenile Least Killifish’s response to harvest depended on how the resource input was allocated between juveniles and adults (Table 1 harvest × food; Fig. 3). In the treatment where the resource input was equally allocated between life stages, stage-independent harvest led to an increase in juvenile biomass. In contrast, juvenile biomass decreased in the treatment where two-thirds of the system productivity was allocated to the adult stage. Adult biomass on the other hand responded with a decrease in all harvest treatments, be it with different rates (Table 1 harvest × food). In conclusion, when resource input was biased towards adults both juvenile and adult biomasses decreased monotonically with increased mortality, whereas juvenile biomass showed an initial overcompensatory increase in biomass with increased mortality with equal resource input.

## Discussion

Most organisms exhibit ontogenetic resource and habitat shifts^19^,^20^,^30^. Consequently, understanding the implications of ontogenetic niche shifts on population and community dynamics is fundamental for the management of natural resources. Our study focuses on the common case with ontogenetic habitat shifts and provides the first long-term, multigenerational experimental evidence that variation in how a given resource input is allocated between life stages within a population affects biomass production in fundamental ways. Specifically, we have shown that allocating a larger part of a fixed resource input to the adult stage more than doubled total population biomass in a consumer population with a more efficient juvenile stage. Intriguingly, this increase in total biomass in a monoculture (one species) situation is similar to the average biomass increase (2.1 mean±0.88 s.d.) reported in studies that focus on the relationship between species diversity and ecosystem functioning, measured as biomass production, by comparing monocultures to poly (several species) cultures^15^,^17^,^32^,^33^,^34^,^35^. Correspondingly, effects of intraspecific variation on ecosystem components as large as or larger than those found for interspecific variation have also been found in a previous short-term, within-generation study^20^. Our results thus show that a change in transfer efficiency and thus biomass production can be induced by only a change in the allocation of a fixed resource input to different life stages without any change in overall resource input, food quality, consumer species or diversity. In contrast, observed biomass increases due to increased species diversity have been attributed to mechanisms such as sampling effects or positive complementarity as a result of differences between species in metabolic types and resource requirements^15^,^17^,^32^,^33^, all of which are characteristics that were kept constant in our experiment. Our study underpins the importance to include life stage variation in studies that investigate ecosystem functional relationships. Therefore, stage-specific system productivity allocation should be included in the list of mechanisms that determine fundamental ecosystem properties such as biomass production, trophic structure and community stability.

The higher biomass occurred with a maturation rate to reproduction rate ratio of one (ontogenetic symmetry), illustrating how a system that is intrinsically asymmetric with respect to the scaling of energy intake rate, maintenance rate and mortality rate can extrinsically be pushed towards ontogenetic symmetry through reallocation of resource input between life stages^18^. Interestingly, our results point to that the highest trophic efficiency occurred for symmetric conditions—the generality of this result needs to be further investigated. This effect of resource allocation on ontogenetic asymmetry within a population explains the difference in population responses to harvest that we observed for the two resource allocation treatments. At equal resource allocation, our population was intrinsically asymmetric and limited by reproduction. This asymmetry is the key mechanism that causes the juvenile biomass overcompensation in response to harvest. The removal of individuals had a strong positive impact on adults’ per capita resource intake and resulted in an increase in overall reproduction rate. Reallocating resources to the adult stage counteracted the intrinsic asymmetry and removed the reproduction bottleneck in the population, so that the scope for stage-specific biomass overcompensation disappeared. Thus, stage-specific resource allocation not only affects population biomass production but also the potential for stage-specific biomass overcompensation to occur at all. Although we chose to keep resource input constant, the same results would be expected if the resource input for juveniles had been kept constant and that for adults had been increased, although the total biomass would have been higher^23^.

Our results have direct practical consequences for the management of exploited populations. For example, juveniles and adults of many fish populations often inhabit different habitats (for example, lake fish that spawn in creeks, anadromous species or marine fish with specific juvenile habitats), and any alteration made to the productivity of one habitat has the potential to affect not only population biomass but also the form of population regulation and thereby population responses to harvest. For example, the generally assumed positive effects of marine-protected areas become less straightforward once life-stage variation in resource allocation within populations is taken into consideration^36^,^37^.

The implications of our empirical results do not stop at the population level. Any alteration of stage-specific biomass production and especially of population regulation has potentially far-reaching implications for higher trophic levels, community structure and stability^23^,^38^,^39^. On the one hand, stage-specific resource allocation will indirectly affect the minimum resource input required for predator invasion if the trophic transfer efficiency is increased. On the other hand, population stage-specific resource allocation will at the same time affect whether a predator can persist at resource input lower than that required for its invasion (emergent Allee effect^40^) that is, whether alternative stable states with catastrophic collapses of predators as a result of stage-specific biomass overcompensation in the consumer are present^23^. Similarly, stage-specific resource allocation will affect whether a stage-specific predator can facilitate the invasion of another stage-specific predator (emergent facilitation^26^). Finally, in systems where juveniles and adults use different resources, an increase in the productivity of the juvenile resource can lead to a counterintuitive extinction of a juvenile-specialized predator^23^.

## Methods

### Summary

**Figure 4:**
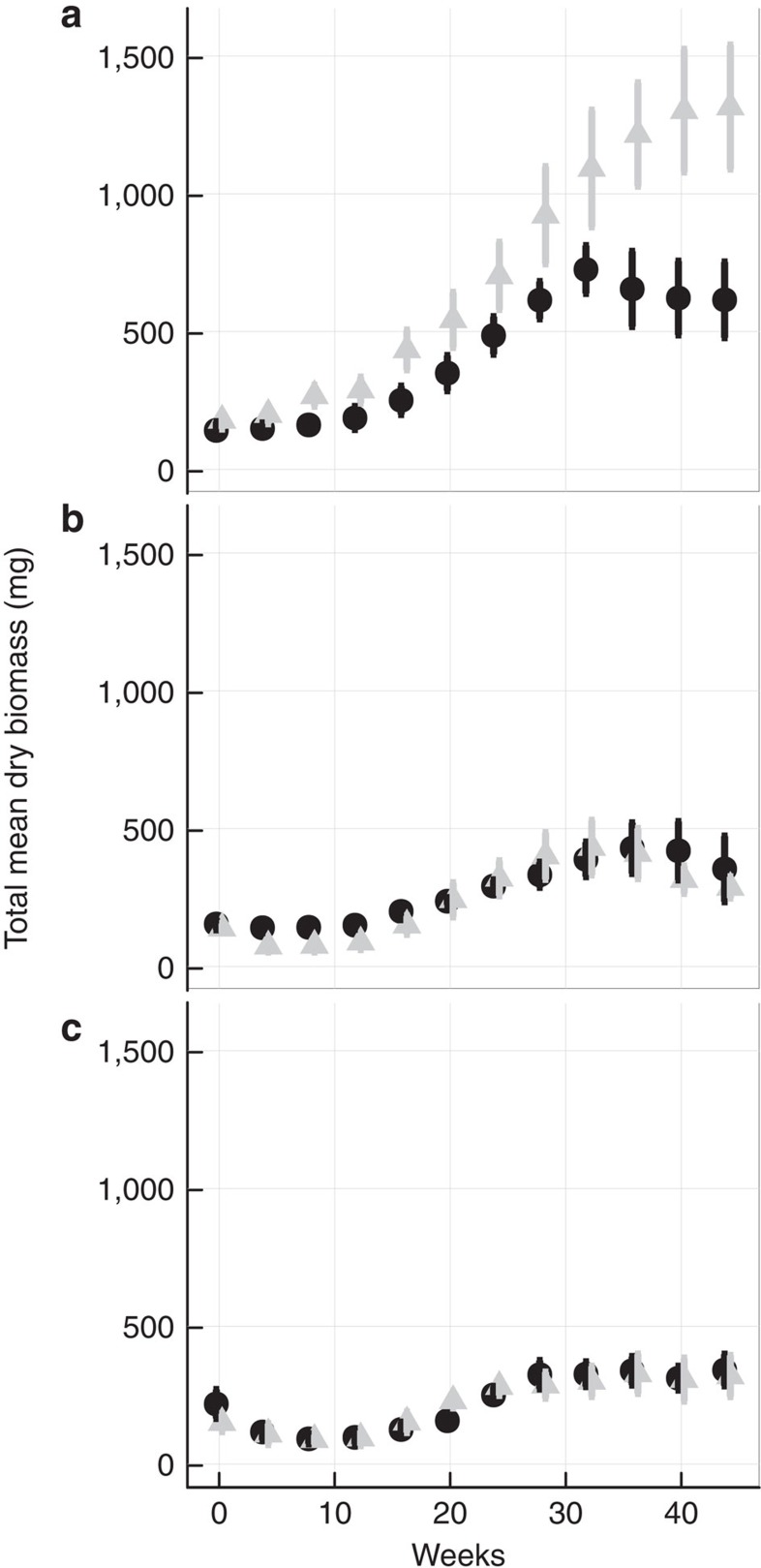
Least Killifish total mean biomass. *H. formosa* mean biomass over the course of the experiment (44 weeks) for non-harvested populations (**a**) and harvested populations (**b**: 0.01 per capita day^−1^; **c**: 0.017 per capita day^−1^). Resource input equally distributed between adults and juveniles (black), and two thirds resource input allocated to the adult habitat (grey). Points represent averages over replicates with vertical lines indicating±1 s.e.m. All treatments were replicated four times.

Juvenile and adult fish (*H. formosa*) were separated between different aquaria to create a juvenile habitat and an adult habitat with distinct resource productivities. Population dynamics were followed over the course of 11 months (six generations), a time period that was sufficient to reach the asymptotic states in the different treatments (Fig. 4). Newborn and maturing juveniles were moved manually between habitats once a week and fortnightly, respectively, yielding estimates of reproduction and maturation rates. Population-wide productivities were kept constant over all treatments (12 × 9.45 mg palletized fish food per day distributed over four feeding events, see below). Two-stage-specific system productivity allocation treatments were applied (**1:1**: equal stage-specific resource productivities and **1:2**: adult habitat productivity twice the juvenile habitat productivity). Productivity allocation treatments were crossed with three-stage-independent per capita harvest levels (0.0, 0.01 and 0.017 per day). Per capita harvest rates were imposed weekly and the number of individuals to be removed were calculated according to the formula *N*_removed_*=N × (*1−e^(−harvest rate × 28 days)^), and removal was distributed as evenly as possible over 4 weeks. All treatments were replicated four times.

### Experimental set-up

*H. formosa* is a viviparous poeciliid fish that occurs naturally in freshwater streams and ponds in North-America in the coastal plains from North-Carolina to Florida. *H. formosa* males grow to a maximum length of 20 mm and females up to 35 mm (ref. 41). Size at birth is 5–8 mm. The generation time is 7 weeks. Males reach maturity at a size of 12 mm and females at a size of 14 mm. Populations used in the experiment were started with 13 females (9 mature), 10 males (4 mature) and 36 juveniles (Table 2), reflecting the equilibrium population structure as determined in (ref. 25). The experiment was executed in accordance with the Swedish law for animal welfare (permit-nr. A41-11).

The experiment was performed in an aquarium system of 56 aquaria (80 l) equipped with air supply, thermostat, bio filters, UV water sterilizer and 15-W neon lights (14-h light/10-h dark regime). Salt was added to prevent infection with ectoparasites (conductivity 900–1,000 μS cm^−1^). All aquaria were equipped with refuges, green plastic thread (Eheim EHFI FIX), loosely packed into four balls per aquarium (two floating and two sunk to the bottom). Water temperature was kept at 25 °C. Water exchange in the aquarium system was constant at 20 l h^−1^.

Feeding was regulated by computer-controlled micro feeders. Populations were fed pelletized food (Dr Basslers Bio Fish Food S) four times a day. In the treatment with equally distributed productivity (1:1), juvenile and adult habitats were assigned the same feeding regime, every second feeding event consisting of one bout (9.45 mg±0.41 (mean±1 s.d.)) and every other feeding event consisting of two bouts (altogether six bouts per stage per day). In the treatment with higher productivity in the adult habitat (1:2), juveniles were fed one bout per feeding event (four bouts per day) and adults were fed two bouts per feeding event (eight bouts per day). In total, each population was fed 12 bouts fish food per day. At the end of a day, no food residues were observed in either the juvenile or the adult habitat, confirming that the population food intake rate was equal to the daily supplied food amount.

At sampling events (every 4 weeks), all fishes were removed from the aquaria, sorted after stage and photographed to later be counted and measured on a computer screen. We used internal standards and sex-specific length–weight regressions to transform measurements into dry weight biomass. During sampling, aquaria were cleaned, and feeders were controlled and refilled.

### Data analysis

In order to capture equilibrium instead of transient conditions, we estimated asymptotic long-term average dry biomass, monthly reproduction and maturation by fitting nonlinear regressions (nls) of the type *y=M+a* × e^(−b × week)^, *y=M* × (*1−e*^(−b × week)^), and *Y=M* with *y=*log(biomass) or *Y=*number of individuals to data from week 12 through week 44 for each replicate (where *M+a* was the intercept, *M* was either the asymptote or simply the intercept and *b* was the initial slope). The value of *M* (or its antilog) represents a measure of the asymptotic long-term average and was extracted for each replicate from the regression model that was identified as the best fit using the Akaike information criterion. Those extracted estimates of the asymptotic long-term average for each replicate were then used as a response variable in two-way analysis of variance analysis with productivity allocation and harvest treatment as explanatory variables. Statistical analyses were performed in R 2.11.1 (ref. 42).

## Author contributions

B.R. and L.P. designed the study, B.R. performed the experiments and data analysis and wrote the manuscript, L.P. and A.M.D.R. contributed to the final manuscript.

## Additional information

**How to cite this article:** Reichstein, B. *et al*. Ontogenetic asymmetry modulates population biomass production and response to harvest. *Nat. Commun.* 6:6441 doi: 10.1038/ncomms7441 (2015).

## Figures and Tables

**Table 1:** Effects of resource allocation and harvest rate on standing biomasses, and maturation and reproduction rates.

	Biomass (mg l^−1^)					
Source of variation	Total		Adult		Juvenile	
Res. Allo.	F_1,18_	8.618**	F_1,18_	8.539**	F_1,18_	0.444
Harvest	F_2,18_	20.746***	F_2,18_	18.369***	F_2,18_	4.561*
Harvest × Res. Allo.	F_2,18_	8.606**	F_2,18_	6.897**	F_2,18_	9.847**

	**Rates (mg l**^−1^ **per month)**			
**Source of variation**	**Reproduction**		**Maturation**	
Res. Allo.	F_1,18_	7.516*	F_1,18_	1.160
Harvest	F_2,18_	3.239	F_2,18_	12.110***
Harvest × Res. Allo.	F_2,18_	2.196	F_2,18_	1.181

ANOVA, analysis of variance; Res. Allo., resource allocation.

F-statistic from two-way ANOVA. All treatments were replicated four times.

*P<0.05, **P<0.01 and ***P<0.001.

**Table 2:** Initial size structure of the experimental *Heterandria formosa* populations.

**Size (mm)**	**Juveniles**	**Females**	**Males**
4–6	8		
6–8	17		
8–10	11		
10–12		1	
12–14		3	6
14–16		3	4
16–18		3	
18–20		2	
20–22		1	
